# Implementing community-based provider participation in research: an empirical study

**DOI:** 10.1186/1748-5908-7-41

**Published:** 2012-05-08

**Authors:** Randall Teal, Dawn M Bergmire, Matthew Johnston, Bryan J Weiner

**Affiliations:** 1 Cecil G. Sheps Center for Health Services Research, University of North Carolina, Chapel Hill, NC, USA; 2 Department of Health Policy and Management, Gillings School of Global Public Health, University of North Carolina, Chapel Hill, NC, USA

**Keywords:** Implementation, Academic-community research partnerships, Cancer clinical trials

## Abstract

**Background:**

Since 2003, the United States National Institutes of Health (NIH) has sought to restructure the clinical research enterprise in the United States by promoting collaborative research partnerships between academically-based investigators and community-based physicians. By increasing community-based provider participation in research (CBPPR), the NIH seeks to advance the science of discovery by conducting research in clinical settings where most people get their care, and accelerate the translation of research results into everyday clinical practice. Although CBPPR is seen as a promising strategy for promoting the use of evidence-based clinical services in community practice settings, few empirical studies have examined the organizational factors that facilitate or hinder the implementation of CBPPR. The purpose of this study is to explore the organizational start-up and early implementation of CBPPR in community-based practice.

**Methods:**

We used longitudinal, case study research methods and an organizational model of innovation implementation to theoretically guide our study. Our sample consisted of three community practice settings that recently joined the National Cancer Institute’s (NCI) Community Clinical Oncology Program (CCOP) in the United States. Data were gathered through site visits, telephone interviews, and archival documents from January 2008 to May 2011.

**Results:**

The organizational model for innovation implementation was useful in identifying and investigating the organizational factors influencing start-up and early implementation of CBPPR in CCOP organizations. In general, the three CCOP organizations varied in the extent to which they achieved consistency in CBPPR over time and across physicians. All three CCOP organizations demonstrated mixed levels of organizational readiness for change. Hospital management support and resource availability were limited across CCOP organizations early on, although they improved in one CCOP organization. As a result of weak IPPs, all three CCOPs created a weak implementation climate. Patient accrual became concentrated over time among those groups of physicians for whom CBPPR exhibited a strong innovation-values fit. Several external factors influenced innovation use, complicating and enriching our intra-organizational model of innovation implementation.

**Conclusion:**

Our results contribute to the limited body of research on the implementation of CBPPR. They inform policy discussions about increasing and sustaining community clinician involvement in clinical research and expand on theory about organizational determinants of implementation effectiveness.

## Introduction

In 2003, the United States National Institutes of Health (NIH) embarked on a fundamental restructuring of the national clinical research enterprise [1]. The centerpiece of its Roadmap for Medical Research has been the intent to create collaborative partnerships between academically-based investigators and community-based physicians to conduct clinical research on a sustained basis [2]. By increasing community-based provider participation in research (CBPPR) through Clinical and Translation Science Awards, federally funded provider-based research networks, and other mechanisms, the NIH has sought to advance the science of discovery by conducting research in clinical settings where most people get their care, and accelerate the translation of research results into everyday clinical practice [1-6]. The Roadmap has spurred discussion of the potential benefits of CBPPR [6-8], infrastructure and workforce training needs for CBPPR [4,9], common barriers to increasing CBPPR [7,10,11], and strategies for overcoming those barriers [7,10].

Missing from this discussion, though, is an empirical investigation of the organizational factors that facilitate or hinder the implementation of CBPPR. For community practice settings, CBPPR is a complex innovation whose implementation requires systemic organizational changes in staffing, workflow, information systems, and reward structures. Even with substantial external assistance, community practice settings may find it challenging to create a supportive organizational context and culture for CBPPR. This task may be especially challenging when community practice settings first become involved in clinical research. Effective implementation of CBPPR during start-up and the early implementation phase, however, may provide a critical pathway for sustained community physician engagement in clinical research.

In this study, we examine the organizational start-up and early implementation of CBPPR in community practice settings. Using case study research methods and implementation theory, we identify the organizational factors associated with the effective implementation of CBPPR in three community practice settings that joined the National Cancer Institute’s (NCI) Community Clinical Oncology Program (CCOP) in the United States, a federally funded provider-based research network with a 28-year history of translating research into practice [12]. Our results contribute to theory about organizational determinants of implementation effectiveness and inform policy discussions about increasing and sustaining community clinician involvement in clinical research.

## Methods

### Conceptual framework

**Figure 1:**
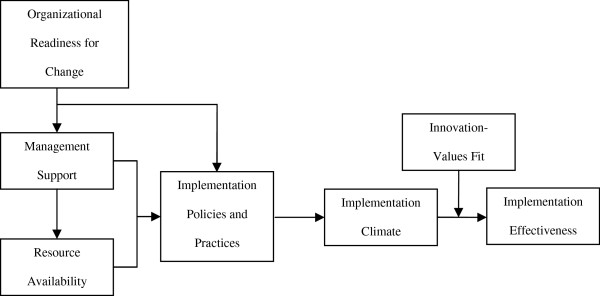
Organizational model of innovation implementation.

We regarded CBPPR as an innovation and employed an organizational model of innovation implementation to guide our study (Figure 1). Briefly, the model posits that consistent, high-quality innovation use (implementation effectiveness) is a function of the organization’s readiness for change, the level of management support and resources available, the implementation policies and practices (IPPs) that the organization puts into place, the climate for implementation that results from these policies and practices, and the extent to which intended users of the innovation perceive that innovation use fosters the fulfillment of their values.

Organizational readiness for change (ORC) is a pre-implementation phase construct that refers to organizational members’ collective confidence to implement change and collective commitment to pursue courses of action that will lead to successful change [13]. Organizational readiness sets the stage for implementation and affects innovation use through other constructs in the model.

Management support and resource availability are two implementation-phase constructs that shape the organizational context of implementation [14-16]. Management support is critical because managers set organizational priorities and control resources needed in implementation. If management supports exists and resources are available, organizational members can put into place more, high-quality policies and practices to promote innovation use [15].

IPPs refer to the plans, practices, structures, and strategies that organizations employ to promote innovation use [15,17]. When IPPs allow organizational members to incorporate the innovation with a significant level of operational, cultural, and strategic fit, there is an increased chance for effective implementation of the innovation. Effective implementation can be achieved through different combinations of policies and practices [16,17]. The collective effect of these policies and practices shape the optimum climate to implement consistent, high-quality innovation use.

Implementation climate refers to organizational members’ shared perception that innovation use is expected, supported, and rewarded [17,18]. Implementation climate emerges from organizational members’ shared experiences with, observations of, and discussions about the organization’s IPPs. Organizations create a positive climate for implementation by employing a variety of mutually reinforcing policies and practices to enhance organizational members’ means, motives, and opportunity for innovation use.

Innovation-values fit (IVF) refers to the extent to which organizational members perceive that innovation use will foster the fulfillment of their values [17]. Although individuals may vary in their values, emphasis here is given to values shared by groups (*e.g.*, oncologists in group-practice). IVF is held to moderate the relationship of implementation climate and implementation effectiveness. Even in a strong implementation climate, innovation use can range from non-use to compliant use to committed use depending on IVF.

### Study setting

**Figure 2:**
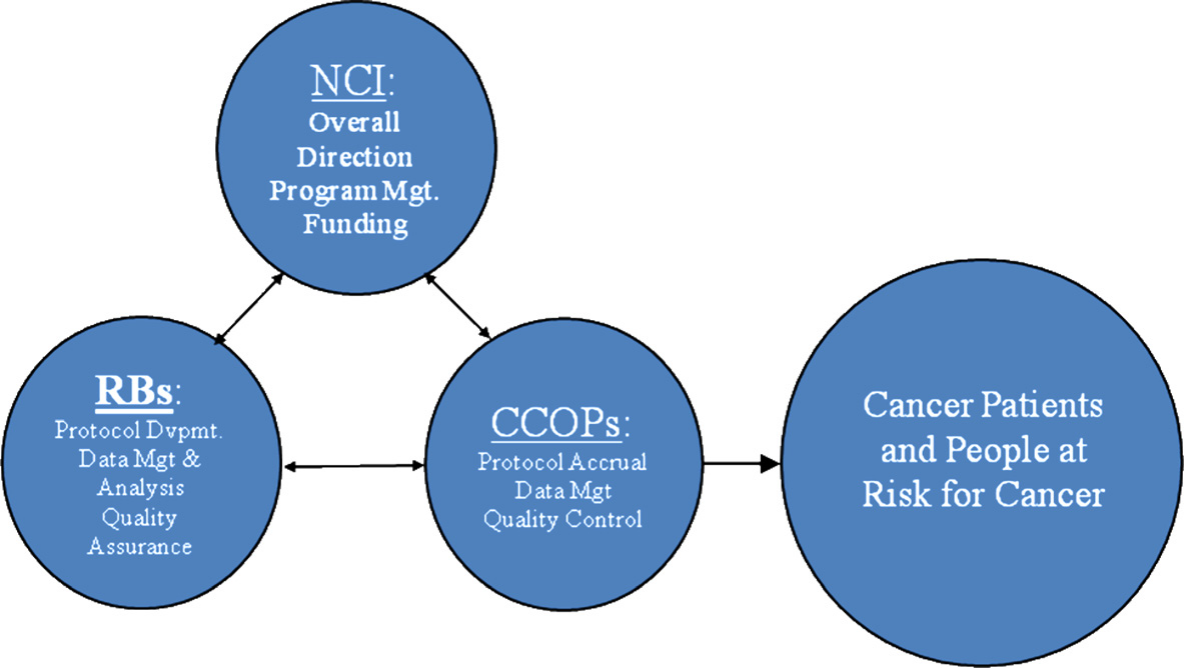
**The National Cancer Institute’s community clinical oncology program.** Adapted from: Kaluzny AD, Morrissey JP, McKinney MM. Emerging organizational networks: the case of the Community Clinical Oncology Program. In SS Mick and Associates, Innovations in Health Care Delivery: Insights for Organization Theory. San Francisco: Jossey-Bass, 1990.

Established in 1983, the CCOP is a three-way partnership involving the NCI’s Division of Cancer Prevention (NCI/DCP), selected cancer centers and clinical cooperative groups (CCOP research bases), and community-based networks of hospitals and physician practices (CCOP organizations) (Figure 2) [12]. NCI/DCP provides overall direction and funding for community hospitals and physician practices to participate in clinical trials; CCOP research bases design clinical trials; and CCOP organizations assist with patient accruals, data collection, and dissemination of study findings. As of December 2010, 47 CCOP organizations located in 28 states, the District of Columbia, and Puerto Rico participated in NCI-sponsored clinical trials. The CCOP includes 400 hospitals and more than 3,520 community physicians.

In FY 2010, the CCOP budget totaled $93.6 million. The median CCOP organization award was $850,000. NCI funds CCOP organizations through a cooperative agreement whereby participating organizations are expected to share the costs with NCI. Continued funding depends on the performance of the CCOP organization in meeting clinical trial enrollment goals (*i.e.*, accrual) set by NCI. CCOP organizations are required to accrue patients in both cancer treatment and cancer prevention and control (CP/C) clinical trials. Cancer treatment clinical trials refer to the evaluation of cancer therapies that generally fall into one of three categories: medical, surgical, or radiation oncology. CP/C clinical trials refer to evaluation of new methods of detecting cancer risk and preventing primary and secondary cancers. It also refers to the evaluation of symptom management, rehabilitation, and continuing care interventions designed to minimize the burden of cancer and improve quality of life. The NCI establishes accrual goals for cancer treatment and CP/C clinical trials based on the CCOP’s past accrual performance and expected accrual performance given the available menu of NCI-sponsored clinical trials.

CCOP organizations are led by a physician Principal Investigator (PI) who provides local program leadership [12]. CCOP staff members typically include an associate PI, a program administrator, research nurses or clinical research associates, data managers, and regulatory specialists. These staff members coordinate the review and selection of new clinical trial protocols for CCOP participation, disseminate protocol updates to the participating physicians, and collect and submit study data. CCOP-affiliated physicians accrue or refer participants to clinical trials, and typically include medical, surgical and radiation oncologists, general surgeons, urologists, gastroenterologists, and primary care physicians. CCOP-affiliated physicians, through their membership in CCOP research bases, also participate in the development of clinical trials by proposing study ideas, providing input on study design, and occasionally, serving in the role of PI or co-PI for a clinical trial.

### Study design

The study followed a hypothetico-deductive approach to qualitative research and used a longitudinal, multiple case study design with the CCOP organization as the unit of analysis. Case study methods are well-suited for studying implementation processes, which tend to be fluid, non-linear, and context-sensitive [19-22]. In addition to permitting in-depth analysis of individual cases, case study methods offer analytic strategies for systematically comparing patterns observed across cases [21,22]. Our sample consisted of three CCOP organizations that are located in the Midwest that received initial CCOP funding between 2002 and 2005. Table 1 shows the characteristics of each of the three CCOP organizations upon inception of the program. An in-depth study of these three CCOPs allowed us to explore the organizational factors that facilitate or hinder the start up and early implementation of CBPPR in community practice settings. The Institutional Review Board (IRB) at the University of North Carolina at Chapel Hill approved this study.

**Table 1:** Characteristics of the CCOP Organizations in Their First Year of Operation

**CCOP** **Site**	**First Year of CCOP funding**	**Number of Performance Sites**	**Number of Accruing Physicians**^**a**^	**Number of CCOP Funded Research Staff FTEs**^**b**^	**Number of NCI-research base affiliates**	**Number of Newly Diagnosed Cancer Patients**
A	2002	3	11	3.7	4	1474
B	2005	7	10	7.2	4	2470
C	2002	2	29	9.3	4	4115

Source: CCOP organizations’ first submitted CCOP progress report.

Note: a. Includes physicians who enrolled at least 1 patient on a treatment and/or CP/C trial.

b. Does not include the PI, co-PI, or CCOP Administrator.

### Data collection procedures

Data were gathered through site visits, telephone interviews, and archival documents from January 2008 to May 2011. A two-person research team visited each CCOP organization between January 2008 and April 2008**.** During the site visit, the team conducted 47 individual and group interviews with CCOP leaders, CCOP physicians and staff, and hospital managers. In subsequent years, the team interviewed the CCOP PI and CCOP administrator from each CCOP organization separately by telephone to gather data about implementation processes, facilitators, barriers, challenges, and opportunities. Table 2 shows the breakdown of interview participants by CCOP organization over time. Each year, the team developed semi-structured interview guides to conduct the interviews, audio-recorded the interviews, and transcribed them verbatim.

**Table 2:** Number and Type of Interview Participants per CCOP Organization, 2008 to 2011

**Site**		**2008 (Yr 1)**	**2009 (Yr. 2)**	**2010 (Yr. 3)**	**2011 (Yr. 4)**
CCOP A	Number	17	5	2	2
	Type	CCOP PI	CCOP PI	CCOP PI	CCOP PI
		CCOP Admin	CCOP Admin	CCOP Admin	CCOP Admin
		2 hospital admin	1 hospital admin		
		6 physicians	2 physicians		
		7 CRAs/support staff*			
CCOP B	Number	11	6	2	2
	Type	CCOP PI	CCOP PI	CCOP PI	CCOP PI
		CCOP Admin	CCOP Admin	CCOP Admin	CCOP Admin
		1 hospital admin	2 hospital admin		
		4 physicians 4CRAs/support staff*	1 physician		
			1 research nurse		
CCOP C	Number	19	6	2	2
	Type	CCOP P I	CCOP PI	CCOP PI	CCOP PI
		CCOP Admin	CCOP Admin	CCOP Admin	CCOP Admin
		3 hospital admin	1 hospital admin		
		2 physicians	3 physicians		
		12 CRAs/support staff*			

* Each site visit included a group interview with clinical research associates (CRAs) and CCOP support staff managing IRB and regulatory issues.

In addition, the research team obtained data from CCOP annual progress reports and grant applications. The NCI requires that CCOP organizations file annual progress reports and periodic re-applications for funding. These documents included detailed data on the CCOP organization’s structure, operations, and accrual data for each treatment and CP/C clinical trial, sorted by CCOP research base, accruing hospital, and accruing physician.

### Analysis

Data analysis involved three phases: data coding, within-case analysis, and between-case analysis. In the first phase, we used qualitative data analysis software, ATLAS.ti 5.0 (and later 6.0), to code the study data. The conceptual framework provided a starting list of codes, which we supplemented with emergent codes as analysis proceeded. Using a common codebook, two investigators (BW, DB) conducted a pilot test by independently coding four transcripts. They then fine-tuned the coding manual’s definitions, decision rules, and examples. Two other research team members (MJ, RT) coded the remaining documents and an investigator (DB) reviewed the coding for accuracy and consistency. The research team coded over 1,000 pages of interview transcripts compiled from the three CCOP organizations.

Due to wide variation in the formatting of progress reports and grant applications across the three CCOP organizations, we could not assign these documents to ATLAS.ti. Instead, we extracted numerical data from these documents (*e.g.*, patient accrual figures for individual physicians) and used reported information to triangulate the results of this study. Over 500 pages of progress reports and grant applications were reviewed.

In the second phase, we conducted a within-case analysis of each CCOP organization. We generated summary reports of each code for each CCOP site in Year 1 through Year 3 of the study. We assessed the degree to which the construct emerged in the data (its ‘salience’) and the degree to which relationships among constructs were consistent with the hypothesized model. Salience in this study refers to the frequency with which constructs (or corresponding codes) appeared in the data and does not necessarily indicate the importance of the construct within the conceptual model [23,24]. For example, the implementation effectiveness construct appeared as a code 39 times in CCOP A interview transcripts, 49 times in CCOP B, and 46 times in CCOP C (Table 3).

**Table 3:** Occurrence of each coded text unit from interviews conducted between 2008 to 2011

**Code**				**Total text units across sites**	**Illustrative quote**
	**CCOP** **A**	**CCOP** **B**	**CCOP** **C**		
**Implementation Effectiveness**	39	49	46	134	So far during this current grant period they’ve only put 11 patients on and they were pretty good about putting about one-third of the patients for the program overall on. So they were good for 30 or 35 [patients].
Organizational Readiness for Change*	44	36	32	112	I had the feeling that they were fairly confident, not overwhelmingly confident but fairly confident that they’d be able to do it [make the minimum requirements to become a CCOP].
**Management Support**	79	54	55	188	Well, we were doing the research and I don’t know if they [hospital management] still know exactly what the CCOP is. They know it’s a grant for our research program. I don’t think there’s a lot of understanding at exactly how much that the research staff does.
**Resources Available**	97	95	68	260	We’re having problems with the trials. We are not—and across the board, the physicians from different areas—the Rad Onc physicians are saying we just don’t have the RTOG trials that we need to be able to work with you all.
**Implementation Policies & Practices**	221	228	237	686	…so we'll see a patient, say we have somebody hat comes in adjuvant colon cancer, and then they potentially could be eligible for N0147. Then we’ll put the information in the chart for the physician with our card and the consent…then we’ll put the guts of the protocol, like the schema and the treatment plan, eligibility in the calendar, and then I have a—what we call pink sheet. It’s a communication sheet that we’ll write out for the physician, and we’ll tell him this is what it looks like this patient might be eligible for, and these are the tests needed, and there’s the consent.
**Implementation Climate**	96	65	83	244	I don’t feel the Rad Oncs are as committed to it, for whatever reason, and it may be because of the way they’re set up and the way they do their work, and it may be set up because of the way their administration is, because we’re a private group. I don’t know for whatever reason why. There's no financial incentive to do it, but I just don’t feel that they’re into it as much.
**Innovation- Values Fit**	51	45	43	139	I think they [physicians] all want to do research. I think they all want to get their patients the highest level of care. I think that in order to participate with the studies that that’s what they’re doing. So they’re looking for the best way to serve their patients, as well as helping with the hospital.
**External Factors**	121	137	120	378	It [economic downturn] has had more of an impact than I would have thought. I was probably naïve about that but patients are much more concerned about their co-pays and about what additional charges they might be facing for things that most of us would consider mundane visits like the infusion room charge when they’re here getting a free drug on a research study. Those things sometimes have been knockout punches for us.
**Total text units over study period**	748	709	684	2141	

*This is a pre-implementation construct only coded during Yr 1 interviews.

In the third phase, we applied the same criteria across the cases to determine if cross-case variation in implementation was consistent with the hypothesized relationships in the model. We generated 12 meta-reports that summarized each code across all three sites from Year 1 through Year 3 of the study. Over 120 summary reports were generated from coded text segments.

## Results

The organizational model for innovation implementation depicted in Figure 1 proved useful for identifying and investigating the organizational factors that facilitated and hindered start-up and early implementation of CBPPR in CCOP organizations. Generally speaking, the model’s constructs were salient in the data (Table 3) and the observed relationships among constructs fit the model. Some constructs exhibited less salience than others, as measured by the number of text units coded for that construct, either because interview questions pertinent to that construct were asked only in specific years (*e.g.*, organizational readiness for change) or because interview participants had less to say about the issue (*e.g.*, management support) or because the construct proved difficult to identify in the natural language responses of interview participants (*e.g.*, IVF). Each year, we asked many questions and heard a great deal about the operational aspects of CBPPR implementation. Not surprisingly, IPP was the most salient construct in our data.

Briefly, results can be summarized as follows. The three CCOP organizations varied in the extent to which they achieved consistency in CBPPR over time and across physicians. All three CCOP organizations demonstrated mixed levels of organizational readiness for change, with strong collective confidence to meet accrual goals but low physician and hospital commitment to CBPPR. Hospital management support and resource availability were limited across CCOP organizations early on, although they improved in one CCOP organization. Through IPPs, all three CCOP organizations provided high levels of research support to some CCOP physicians, but not others. However, none created formal expectations or accountability for physicians to help the CCOP organization meet its accrual goals. Further, all three organizations used relatively weak IPPs to recognize and reward physicians for enrolling patients in clinical trials. As a result, all three created a weak implementation climate. Patient accrual became concentrated over time among those groups of physicians for whom CBPPR exhibited a strong IVF. Several external (environmental) factors also influenced innovation use, complicating and enriching our intra-organizational model of innovation implementation.

Below we describe our results in detail, beginning with implementation effectiveness.

### Implementation effectiveness

As an organization-level construct, implementation effectiveness refers to the consistency and quality of aggregate innovation use among intended innovation users. We operationally defined innovation use as patient accrual, or the enrollment of new patients in NCI-sponsored clinical trials. Aggregate innovation use is measured in terms of CCOP-level patient accrual.

**Figure 3:**
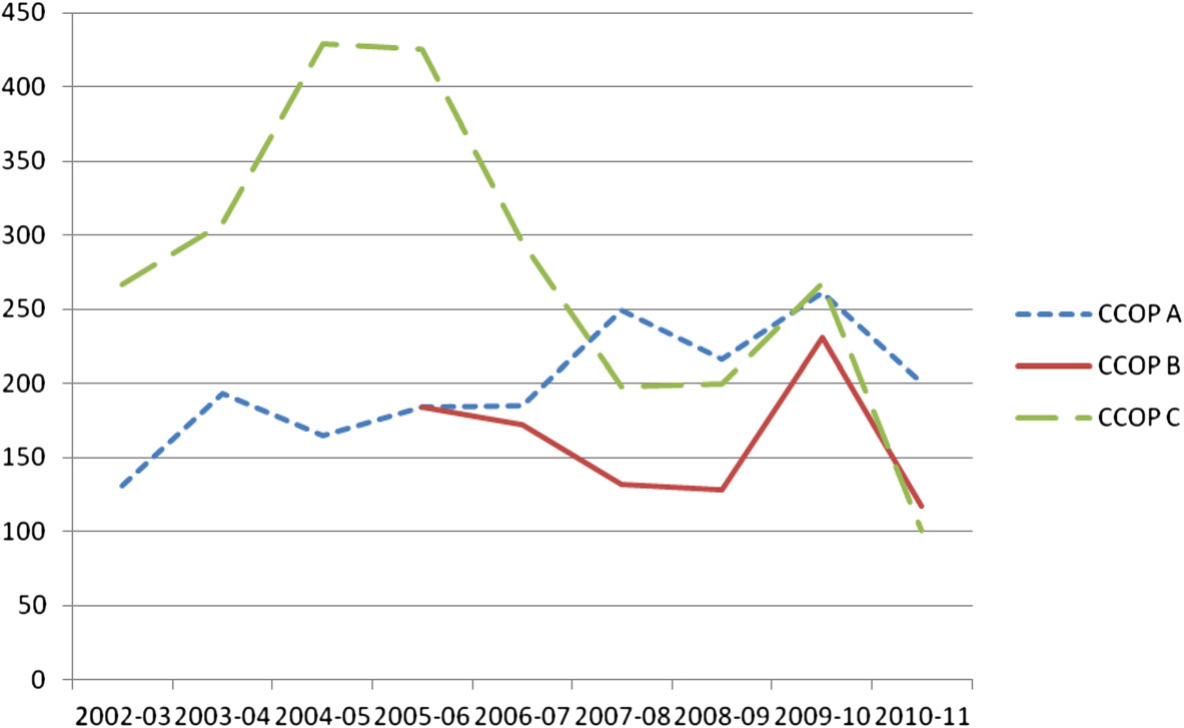
**Total patient accrual by CCOP Organizations.** Source: The CCOP, MBCCOP, and Research Base Management System website: https://ccop.nci.nih.gov/Note: Total patient accrual includes all patients enrolled in NCI-sponsored cancer prevention and control trials and NCI-sponsored cancer treatment trials. CCOPs report patient accrual to the NCI on a 12-month basis from 1 June to 31 May.

Figure 3 shows that CCOP A exhibited the greatest consistency over time in total patient accrual to NCI-sponsored clinical trials (*i.e.*, treatment and CP/C accrual combined). CCOP C exhibited a substantial jump in total patient accrual in the first three years of CCOP funding, followed by an even larger decline. CCOP B exhibited a similar, but less pronounced pattern. All three CCOP organizations struggled with patient accrual in 2010 and 2011 due, in part, to external factors (described below). Viewed somewhat differently, CCOP A met the NCI’s accrual goals in eight of the nine observed years for CP/C trials and six of the nine observed years for treatment trials. CCOP C met the NCI’s accrual goals in eight of the nine observed years for CP/C trials, but only two of the nine years for treatment trials. CCOP B met the NCI’s accrual goals in two of the six observed years for CP/C trials and none of the six years for treatment trials. In sum, the consistency of aggregate innovation use was highest in CCOP A, then CCOP C, then CCOP B.

The quality of aggregate innovation use did not vary meaningfully across the three cases. There were minor audit issues reported around eligibility criteria and protocol selection in all three cases, but the data quality and research procedures were generally robust.

The three CCOP organizations also varied in the consistency with which physicians engaged in CBPPR. To examine how and why this is so, we turn to our organizational model of innovation implementation, beginning with an analysis of organizational readiness for change.

### Organizational readiness for change

Success in the start-up phase of implementation depends in large part on ORC. As noted earlier, ORC concerns organizational members’ collective confidence to implement change and collective commitment to pursue courses of action that will lead to successful change. In all three CCOP organizations, collective confidence was high. Prior to becoming a CCOP, all three CCOP organizations had participated in NCI-sponsored clinical trials as research affiliates of cooperative groups. As research affiliates, they gained experience enrolling and managing patients on clinical trials and used the per-case reimbursement that they received for enrolling patients to hire research staff to support participating physicians.

Interview participants said that, when they applied for CCOP funding, they knew they would have to significantly ramp up their clinical trials research operations to meet the NCI’s accrual goals. Moreover, they acknowledged some trepidation about having to enroll patients in CP/C trials, a type of clinical research with which they had less experience. Nonetheless, they felt confident that they could quickly increase the scope and scale of their research operations:

‘We were pretty confident we’d be able to meet the treatment trials [accrual goal], if the right trials were open…. The prevention [trials accrual goal] has always been kind of an iffy in the very beginning, just because there weren’t [large prevention] trials available…. There wasn’t a SELECT [the Selenium and Vitamin E Cancer Prevention Trial], there wasn’t a STAR [Study of Tamoxifen and Raloxifene]. So we thought we’d be able to meet the treatment. We never knew about the prevention.’—CCOP B, CRA group interview.

Although collective confidence was high, collective commitment was more variable in all three CCOP organizations. Some physicians were committed and enthusiastic about becoming a CCOP organization; other physicians were ‘supportive’ or merely ‘interested.’ In retrospect, it is not clear whether physicians understood that, as a CCOP, they collectively would be ‘on the hook’ for a much higher level of accrual than they were used to generating as a research affiliate. To meet the NCI’s expectations, physicians would have to step up their personal participation in the clinical research, enrolling patients routinely rather than occasionally in clinical trials. The CCOP PIs of all three CCOP organizations succeeded in building physician support to become a CCOP organization, but the broad support they garnered did not translate into broad (or deep) commitment to enrolling more patients in clinical trials after the CCOP grant was awarded:

‘I think that they [physicians] were tolerant of it but I don’t know that they would have independently thought of it and I don’t know that they perceived that to become a CCOP was going to move us to a different level or establish a different regard for our practice in the community. But, you know, I do think that they heard me out and I think they were supportive of it when they heard the reasons I felt it was a good idea.’—CCOP B, PI

In addition, it is not clear that the CCOP PIs made the strategic or competitive case to hospital managers for becoming a CCOP organization (or, if they did make the case, that hospital managers understood it). In none of the cases did the decision to apply for CCOP funding come from hospital managers. Instead, the PIs came up with the idea to apply and wrote the CCOP proposal. In one case, the PI waited until a ‘friendly’ but interim hospital manager was in place before asking the hospital to sign-off on the CCOP proposal. While the PI received the sign-off, the stage was set for low levels of management support once the program was funded.

In all three cases, the minimal involvement of hospital management in the decision to apply for CCOP funding set the stage for minimal management support once the grant was awarded. In all three cases, managers viewed the CCOP as a ‘doctor’s program,’ with potential benefits or advantages for the hospital either misunderstood or unrealized:

‘For many years here, they weren’t supportive. Then they became supportive when they thought that it was [good] for the hospital’s existence. So, hospital administrators will not support programs that are for research, and maybe that was my wrong approach because I told them this was a research project. In retrospect, what I should have done is said this is not a research program, this is a quality of care program.’—CCOP C, PI

In sum, the three CCOP PIs overestimated collective confidence and underestimated collective commitment. Although all three CCOP organizations met NCI’s accrual goals right away, the mixed organizational readiness for change exhibited in the pre-implementation phase foreshadowed the problems that all three cases encountered later in implementation.

### Management support and resource availability

Management support matters because implementation is resource intensive. Managers can support CBPPR by allocating needed financial, human, and material resources. In addition, they can shape the implementation context by communicating that CBPPR is an organizational priority, encouraging organizational members to implement policies and practices to support CBPPR. Finally, managers can support implementation by overcoming resistance to change and resolving disputes over resource allocations, organizational routines, and lines of authority.

Given the minimal involvement of hospital managers in the decision to become a CCOP organization, management support during implementation was largely symbolic. The managers we interviewed expressed verbal support for the CCOP organization, but did not see CBPPR as important in advancing the hospital’s strategic goals or improving its competitive position:

‘When I attract physicians to the organization, there’s eight or nine things that come to the top and this [the CCOP] would probably be one of I’d say five or six, as opposed to the first five. So, I would tell you it’s not in my top three. I would say that when I’m talking about attracting doctors to the organization, I’m generally talking more at the global level.’—CCOP B, hospital manager.

In two cases, hospital managers made no financial commitment to CBPPR over the course of our study, despite the fact that NCI funds CCOP organizations with expectations that institutional cost-sharing will occur. Instead, managers expected the CCOP organization to ‘live on its CCOP grant’ and any supplemental funding that physicians generated from participating in pharmaceutical industry trials:

‘Basically, it amounts to the CCOP grant, we get a little bit of money from the cooperative groups and we get the money from industry trials and the hospital helps in the sense that they own the foundation that gives us some philanthropic support that helps us fill the space between what we can generate and what our true costs are…they’re [the hospital] kicking in zero dollars for every dollar that we generate.’—CCOP A, PI

In the third case, hospital managers realized that CBPPR had strategic value in light of the hospital’s plans to become a major teaching affiliate of a newly opened medical school. Three years into implementation, hospital managers began making substantial financial commitments to the CCOP organization, starting at one million dollars and, later, as the Great Recession continued, reducing the amount to $800,000:

‘I think it’s evolved through the years, but I think research has taken a greater importance…as that has happened people have seen, well, you have the CCOP, it’s been the gem, it’s been sitting there all these years and it’s just kind of like rediscovered every year. But now, like I said, with the medical school, the importance of research is clearer.’—CCOP C, physician

Although management support increased over time in one case, the modest management support that existed in all three CCOP organizations during start-up and early implementation was, in interview participants’ minds, both a blessing and a curse. On the one hand, the CCOP organization could ‘fly under the radar’ with little management interference. Moreover, it was largely shielded from the staffing reductions and budget cuts that the hospital imposed elsewhere as the ‘Great Recession’ persisted. On the other hand, the CCOP organization could not expand its operations beyond what the CCOP grant could sustain. Further, the CCOP organization could not use hospital managers’ authority and resources to implement policies and practices that would make physicians accountable for helping the CCOP organization meet its accrual goals.

### Implementation policies and practices

Organizations can make use of a wide variety of IPPs to enhance organizational members’ means, motives, and opportunities for innovation use. IPPs are cumulative, compensatory, and equifinal [15,17]. That is to say, all other things being equal, the more IPPs an organization employs to support innovation use the better. However, high-quality IPPs can sometimes compensate for missing or poor-quality IPPSs. For example, excellent in-person training could substitute for mediocre program manuals. Finally, different combinations of IPPs can produce the same results.

Our analysis identified three IPP themes. First, all three CCOP organizations provided a high level of staffing and operational support for some, but not all, of the physicians who wanted to enroll patients in NCI-sponsored clinical trials. Although the specific IPPs used by the three CCOP organizations differed somewhat (*e.g.*, some used a mix of registered nurses and clinical research associates while others used only registered nurses to staff the clinical trials program), all three provided support by screening charts to identify potentially eligible patients, flagging charts to prompt physicians to introduce trials to eligible patients, educating and consenting interested patients, following patients once enrolled in a study, and managing IRB and other regulatory requirements. However, they could only provide such high levels of support to those physician practices located ‘on-site’ or in close proximity to the hospitals where research staff worked. Support for ‘off-site’ physician practices was harder to provide or sustain due to travel time and logistical difficulties. The limited support CCOP organizations could offer these physicians contributed to uneven distribution of patient accrual.

Second, all three CCOP organizations avoided using IPPs to create formal expectations or accountability for physicians to help the CCOP organization meet its accrual goals. The CCOP PIs and CCOP administrators routinely cajoled, sometimes exhorted, and occasionally begged physicians to enroll more patients in clinical trials. They regularly provided physicians with feedback on the CCOP organization’s accrual performance relative to NCI’s accrual goals and usually provided physician-level patient accrual figures. Yet, none were willing to put into place a policy specifying the minimum number of patients per year that a physician had to enroll in order to maintain membership in the CCOP organization and, thereby, enjoy the reputational benefit of having an ‘NCI affiliation’:

‘This institution will not tell the referring doctors that they can’t be members of the CCOP for some criteria. You’ve got to put, let’s say, ten patients on a year. In fact, the institution is going to the opposite direction.’—CCOP C, PI

Moreover, none were willing to confront physicians who maintained their membership in the CCOP organization, yet consistently contributed no patient accrual. Interview participants mentioned several reasons why they were unwilling or unable to implement such policies and practices, including not wanting to shut anyone out who might at some point contribute accrual; not wanting to exclude off-site physicians who received little or no support from the CCOP organization; and not wanting to confront colleagues who, in some cases, were business partners:

‘No, the group has to provide support for the clinical research program but there’s no individual expectation that every physician will accrue ten patients per year to clinical trials. We keep trying to bang that drum and we provide feedback to our doctors on a regular basis about how they are doing, but I can’t tell that they have the requisite shame that I would hope that they would have with some of the numbers.’—CCOP B, PI

CCOP organizations sought to compensate for these missing policies and practices by providing more research support, but with little success.

Finally, all three CCOP organizations used relatively weak IPPs to recognize and reward physicians for enrolling patients in clinical trials. Financial incentives or rewards were out of the question due to ethical, legal, and regulatory concerns. So, CCOP organizations fell back on social recognition and non-monetary rewards, neither of which remained effective over time. Social recognition included verbal praise in meetings, certificates of appreciation, complimentary write-ups in newsletters, and token awards given to the highest-accruing physician. Non-monetary rewards included paid travel to conferences, batches of cookies, and other small gifts. The motivating effects of these rewards diminished over time:

‘I think that we’ve tried all the rewards that probably many other research organizations have tried. You know, we’ve given, boxes of candy and we’ve given gift certificates and cookies…we found our doctors are not going to enter patients on clinical trials for the benefit of winning the chocolate chip cookies or winning a gift certificate.’—CCOP B, PI

In sum, all CCOP organizations employed IPPs unevenly. Although some IPPs can substitute for other IPPs, the challenges that the CCOP organizations experienced providing support to off-site physicians and the minimal or low-power IPPs that they used create expectations, accountability, recognition, and rewards produced a weak implementation climate.

### Implementation climate

IPPs influence innovation use through implementation climate. Organizational members ascribe meaning to the IPPs that they experience directly or vicariously. Through experience, observation, and discussion, they develop a shared sense of whether innovation use is expected, supported, and rewarded. This shared sense is important when the organizational benefits of innovation use depend on consistent, high-quality use by many organizational members. Such is the case with CBPPR in CCOP organizations. The NCI evaluates and funds CCOP organizations based on aggregate (*i.e.*, CCOP-level) accrual.

Given the uneven IPPs that CCOP organizations put into place, implementation climate was weak in all three cases. None of the interview participants reported that they were expected either by the CCOP organization or by hospital management to enroll any particular number of patients in clinical trials each year to maintain membership in the CCOP organization. Likewise, none reported that they were recognized or rewarded in a meaningful way for helping the CCOP organization meet its accrual goals. Physicians practicing in or near the hospital felt supported by the CCOP to enroll patients in clinical trials; off-site physicians did not.

Although implementation climate was weak in all three CCOP organizations, all three nonetheless met or exceeded NCI’s accrual goals. To understand how the CCOP organizations achieved this feat, we must examine the pattern of IVF among physician groups.

### Innovation-values fit

As noted above, IVF refers to the extent to which organizational members perceive that innovation use will foster the fulfillment of their values. Values are concepts or beliefs that pertain to desirable end states or behaviors. Although individuals can hold different values, so too can groups. Different physician groups, for example, may hold different beliefs about clinical trials participation. Individuals and groups can also differ in the amount of feeling they attach to a concept or a belief. Clinical trials participation might be valued strongly and considered a priority, or it might be valued weakly and considered desirable, but not necessary.

When implementation climate is weak, innovation use depends primarily on IVF. Individuals and groups that strongly value CBPPR will enroll patients in clinical trials even if they perceive little expectation or extrinsic reward for doing so, provided adequate support exists. If inadequate support exists, ‘true believers’ will find it difficult to enroll patients on a consistent basis, leading perhaps to frustration and disappointment. By contrast, individuals and groups that do not strongly value CBPPR will enroll few patients, if any, because they see little reason to do so when enrolling patients is neither expected, nor supported, nor rewarded.

We observed individual differences in IVF in all three cases. Not surprisingly, the CCOP PIs highly valued CBPPR and enrolled many patients. In one CCOP organization, a physician enrolled many patients in pharmaceutical industry trials but relatively few in NCI-sponsored clinical trials. Industry trials exhibited a greater fit with his high-intensity values because, in his view, they gave him access to newer, more exciting drugs. In another CCOP organization, a physician stated that he did not like the NCI’s treatment trials because they were not, in his view, always addressing important scientific questions; however, he was willing to help the CCOP organization achieve its accrual goals by enrolling patients in cancer control trials.

We also observed group-level differences in IVF in all three cases. Medical oncologists as a group more strongly valued CBPPR, and therefore enrolled more patients in NCI-sponsored clinical trials, than did radiation oncologists or other physician groups. Interview participants in two CCOP organizations noted that radiation oncologists as a group were ‘supportive,’ but not ‘committed’ to enrolling patients in NCI-sponsored clinical trials. The radiation oncologists would follow the clinical trial protocol for those patients that the medical oncologists enrolled, but they would not enroll patients in radiation-therapy clinical trials in sufficient numbers to help the CCOP organization meet its accrual goals:

‘The total again is 8% of the patients. So that means they put eight, nine, ten patients on this whole year…I’ve been here for 20 years and this is pretty typical for the way the radiation therapists have performed over the last 20 years. This is nothing different. They just don’t do a lot of randomized, you know, Phase III trials, which is what we do as a CCOP. They do a lot of Phase II trials.’—CCOP C, PI

Even when provided with additional research staff support, radiation oncologists as a group would not contribute much on a consistent basis. CBPPR was simply not a high-intensity value for them, interview participants said.

In a third case, radiation oncologists as a group strongly valued CBPPR, as evidenced by their active in-house research program. However, they preferred to enroll patients in in-house studies rather than NCI-sponsored clinical trials because such studies were more instrumental in fulfilling their values (*i.e.*, they felt such studies were more interesting and easier to do).

Given weak implementation climate and variable IVF, all three CCOP organizations exhibited inconsistent, uneven distribution of patient accrual among individuals and groups of physicians. Of the three cases, CCOP A exhibited the strongest IVF among medical oncologists as a group. This CCOP organization recruited and hired physicians who valued CBPPR, and it reinforced CBPPR as a group value in a variety of formal and informal ways. Reflecting a more distributed (or even) pattern of patient accrual, the PI of CCOP A enrolled on average only 28% of the patients per year that CCOP A accrued to NCI-sponsored clinical trials from 2002 to 2010. By comparison, accrual was more concentrated at CCOP C and CCOP B. In the former case, the average annual accrual contribution of the PI was 34%, while in the latter case it was 44%. At start-up, CCOP C exhibited more evenly distributed accrual among physicians, with the CCOP PI accounting for only 18% of patient accrual in the first year. With off-site physicians receiving little support, and radiation oncologists focused on in-house studies, initial enthusiasm faded and accrual become increasingly concentrated among the few ‘true believers.’ By 2008, six years after start-up, the CCOP PI’s contribution accounted for over 42% of total patient accrual. In CCOP B, the concentration of patient accrual was high at start-up and increased steadily over time. In the first year, the PI’s contribution accounted for 39% of total patient accrual. By 2009, it amounted to 59%, despite personal appeals from the highly regarded PI and increased research support for low- or non-accruing physicians. An interview participant described the situation:

‘And our accruals here are actually up but once again, we’re really concerned that [the CCOP PI] has [put] 40 some odd patients on treatment trials. Everyone else in the CCOP is at single digits. And we don’t know, we’re getting ready to have a discussion with the other physicians and [the PI] and I are mulling things over on how to present this but this can’t go on. It just can’t go on. If you want us to maintain ourselves as a viable CCOP, everybody’s got to pull their weight.’ –CCOP B, CCOP Administrator

### External factors

Although the organizational model of innovation implementation used in this study focused our attention on intra-organizational facilitators and barriers, we observed that CCOP organizations are heavily dependent on two external resources needed for CBPPR—trials and patients. In varying degrees, all three CCOP organizations experienced difficulties obtaining these resources, which, in turn, exacerbated the challenges posed by a weak implementation climate and variable IVF.

Interview participants noted that the menu of NCI-sponsored clinical trials available to CCOP organizations has shrunk in the past two years and contains significant gaps in common disease areas, such as colon cancer. Interview participants attributed the trial availability problem to the consolidation of the cooperative group system that is currently taking place in response to an influential, yet critical report by the Institute of Medicine [25], a not-for-profit, non-governmental organization that advises the federal government on issues related to biomedical science, medicine, and health. With fewer treatment trials available, CCOP organizations are struggling to meet their treatment trial accrual goals. In addition, with no large cancer prevention trials available, CCOP organizations are trying to meet their CP/C accrual goals through symptom management trials. These smaller trials often close quickly to new patient accrual, disadvantaging those CCOP organizations (like CCOP C) that work with slow IRBs, which must approve studies locally before CCOP organizations can enroll patients in them.

Interview participants further noted that NCI-sponsored clinical trials are increasingly testing targeted therapies applicable only to small subgroups of cancer patients. Not long ago, breast cancer treatment trials would open to almost any patient with, say, advanced disease and no co-morbidities. Today, trials are testing different therapies for node positive and negative breast cancer, which can be further divided into pre- and post- menopausal categories, which can be further sub-divided into other categories, creating approximately 16 different groups of breast cancer patients. Similar trends are occurring in treatment trials for other cancers. Interview participants referred to this as a ‘needle in a haystack’ problem:

‘What’s happening is [that] what used to be gigantic baskets of patients are [now] being divided up by biology. It’s better medicine to divide them…. Each one of these breast cancers is a bit different, [and] each one of them has a different [treatment] protocol. So now…I just cut breast cancer into 16 different groups…. And I think that accounts for a large degree why we don’t really have any rainmaker studies.’—CCOP A, PI

To meet their accrual goals, some interview participants observed, they cannot afford to miss any opportunity to enroll an eligible patient. Hence, they feel frustrated when low- or non-accruing physicians let an eligible patient go un-approached.

Finally, interview participants commented that meeting the NCI’s expectations for patient accrual became increasingly difficult in the face of persistent economic recession and increased market competition. Midwestern states, where the three COCP organizations operate, have been hit especially hard. Patient volume has declined across the board as patients put off medical care to manage other living expenses. Interview participants noted that patients have voiced more concern about enrolling in a clinical trial because they are worried about their health insurance coverage and out-of-pocket costs. Given job insecurity, patients are more reluctant to take time off work to receive multiple rounds of cancer treatment and return for follow-up clinical visits. At the same time, local market competition has grown. The PI of CCOP A noted, for example, that he and his four partners were the only medical oncologists in town in the first year of the CCOP grant. Now, he has eight partners and there are two competing oncology practices not affiliated with the CCOP organization that have seven more medical oncologists. The number of cancer patients has not increased threefold over time.

## Discussion

CBPPR is a promising strategy for translating research results into clinical practice. By engaging providers in the research process, researchers can gain insight into the clinical issues in community practice settings, obtain provider input on study design and implementation concerns, and discover the tacit practice-based knowledge that exists in community practice settings. By doing research in their own practice settings, providers may feel a greater sense of ownership and trust in clinical research results, which, in turn, may increase their commitment to acting on research findings. Yet, CBPPR does not occur spontaneously or effortlessly. To participate in research, community-based providers often must implement systemic changes in organizational staffing, office workflow, information systems, and reward structures. In addition, they must develop an organizational culture (shared values) that supports CBPPR and evidence-based practice.

Our study findings contribute to the limited body of research on the implementation of CBPPR. Three findings in particular have policy and practice implications that merit discussion. First, CBPPR is difficult to create and sustain in the absence of management support from local, affiliated hospitals and health systems. Managers not only control critical institutional resources, but play (or could play) an important role in legitimizing the value of CBPPR, authorizing policies and practices to support CBPPR implementation, and addressing turf issues and other political obstacles to CBPPR. From a policy standpoint, the NIH and other federal agencies seeking to increase CBPPR should require hospital and health system executives to make tangible institutional commitments in order to participate in federally funded provider based research networks or other programs. In its National Community Cancer Centers Program, the NCI went a step further by engaging the chief executive officers of participating hospitals and health systems as a stakeholder group, inviting them to national program meetings, recognizing their participation publicly, and creating opportunities for them to meet privately as a group with the NCI Director [26]. From a practical standpoint, community-based providers need to make the strategic or business case for CBPPR by showing hospital and health system executives how participating in clinical research aligns with and advances organizational priorities. It might be useful to frame CBPPR as ‘quality enhancement’ rather than ‘research,’ as the CCOP PI did in the one case we observed where hospital managers made a significant financial commitment to CBPPR. Newly available tools for assessing the business case could also prove useful [27].

Second, community-based providers face demanding schedules and heavy workloads and, thus, need substantial staff support to participate in clinical research. CCOP organizations struggled to provide adequate staff support to physicians who practiced in locations more than a few miles from the hospital or physician office buildings where research staff were based. Even high-accruing physicians found it difficult to enroll patients in clinical trials when they traveled to outlying rural clinics. The policy implication is that the NIH and other federal agencies seeking to promote CBPPR should be prepared to provide adequate, consistent funding to create and maintain the research infrastructure necessary to support CBPPR. To its credit, the NCI uses peer-reviewed cooperative agreements to award multi-year grant funding directly to CCOP organizations, rather than passing the money through academic medical centers or other intermediaries [12]. Direct funding puts more resources in the hands of community-based providers who want to engage in clinical research. Multi-year funding offers predictability and stability for longer-term planning, organizing, and staffing. Cooperative agreements permit local flexibility in resource allocation, while ensuring regular performance monitoring and financial reporting. From a practical standpoint, community-based providers could stretch the resources they have for CBPPR by experimenting with decentralized research staffing models, having a research staff member accompany traveling physicians, training office staff members to assist with research tasks, using information and communications technologies to support providers’ participation in research, and triaging research staff to support those ‘off-site’ providers with the greatest potential for and likelihood of increased research participation.

Finally, research infrastructure is necessary, but not sufficient to gain providers’ commitment to CBPPR. Although CCOP organizations were able to provide high levels of staff support to at least some CCOP physicians, they did not succeed in creating a shared sense among CCOP physicians that enrolling patients in clinical trials was expected, supported, and rewarded. Given a weak implementation climate and variable degrees of IVF, all three CCOP organizations exhibited inconsistent, uneven distribution of patient accrual among individuals and groups of physicians. Implementation climate is more amenable to policy and practice intervention than IVF [16,17]. At the policy level, the NIH, other federal agencies, and professional associations could stimulate demand for CBPPR by launching a national public service advertising campaign to encourage patients to participate in clinical research [25]. Increased patient demand could increase clinicians’ sense that CBPPR is expected. Further, these parties could do more to provide public recognition of clinicians who engage in CBPPR. On a practical note, community-based providers should consider establishing formal expectations and accountability procedures to strengthen the implementation climate for CBPPR [25]. The Delaware Christiana CCOP organization did so in 2008 [28] and later reported that CCOP accrual increased, despite one physician’s resignation from the CCOP organization [29]. In 2011, two of the three CCOP organizations we studied followed Delaware Christiana’s example. It is too soon to tell what results they achieved.

The organizational model of innovation implementation employed in this study proved useful for investigating the organizational factors that facilitated and hindered start-up and early implementation of CBPPR in CCOP organizations. However, study findings results suggest two ways in which the model could be refined. First, the model’s focus on intra-organizational factors obscures the extent to which extra-organizational factors influence innovation implementation within organizations. Community-based providers are heavily dependent on external resources and subject to many legal, regulatory, and professional constraints. While these external factors may influence innovation implementation through intra-organizational factors identified in the theory (*e.g.*, by shaping management support, resource availability, and IPPs), they could also directly influence innovation use. CCOP organizations, for example, cannot modify the protocols of NCI-sponsored clinical trials once the trials become open for patient accrual. CCOP organizations can decide not to make use of trials that do not fit local patient populations, practice patterns, or organizational resources. Doing so, however, limits CBPPR implementation and affects CCOP accrual. External factors may also change the conceptual meaning of intra-organizational factors. For CCOP organizations, for example, resource availability includes both organizational slack—that is, unused and potentially available financial resources from hospitals [15,30-32]—as well as CCOP grant funding.

Second, we described IPPs as cumulative, compensatory, and equifinal. Specifically, following Klein *et al.*[15,17], we argue that the presence of some high-quality IPPs may compensate for the absence or low quality of other IPPs. Although we found some support for this idea, our results suggest there are limits to IPPs’ substitutability. Providing more research support to low- or non-accruing physicians, for example, did not compensate for missing or weak IPPs concerning expectations, accountability, recognition, or rewards. Organizational members must have not only the means to achieve consistent, high-quality innovation use, but also the motives and the opportunity to do so. It may be the case that IPPs can compensate or substitute for one another within the categories of means, motives, and opportunities, but not perhaps across these categories.

### Study limitations

All studies have limitations. Ours is no exception. Case study research emphasizes depth over breadth, insight over generality [20-22]. Three cases, no matter how closely studied, do not provide a sufficient basis for statistical generalization. To help others assess the reasonableness of applying our findings to other practice-based research networks (PBRN), we offer a ‘proximal similarity model’ [33] that suggests two important gradients along which PBRNs vary: the type of clinical research conducted by the PBRN, and the strength of the science upon which it is based; and the level of federal support provided to the PBRN. The more proximal a PBRN is to the CCOP on these gradients, the greater the reasonableness of applying study findings beyond the specific people, places, settings, and times from which they were generated. With this proximal similarity model in mind, two factors increase our confidence in the transferability of our study findings. First, the CCOP has already served as a model for other federally supported PBRNs [34], and the NIH continues to look to the CCOP for guidance as it implements the Roadmap. Second, a recent survey found that many clinical research networks funded by the NIH share with the CCOP key organizational features with respect to funding, design, and operation [35]. This means that many NIH-funded clinical research networks are proximally similar to the CCOP.

As is true of all research, case study research involves an irreducible element of expert judgment. We employed several case study research tactics to increase the dependability and credibility of our study results, including the use of theory to conceptually and operationally define constructs; use of multiple sources of data; use of experienced interviewers; use of a common interview guide and codebook; audio-recording and transcription of interviews; use of software to code transcripts and document coding decisions; and duplicate coding and systematic review of coded transcripts, summary reports, and meta-reports by multiple research team members. We feel confident in our use of these time-honored case study research tactics, but we cannot discount fully the possibility that investigator bias in interpretation influenced our results.

## Conclusion

In conclusion, a small, but growing body of research indicates that community-based providers who engage in clinical research more rapidly adopt evidence-based clinical services than those who do not engage in clinical research [36-41]. These studies suggest that CBPPR holds promise as a strategy for accelerating the translation of research results into clinical practice. Our results contribute to the limited body of research on the implementation of community-based provider participation in research. They inform policy discussions about increasing and sustaining community clinician involvement in clinical research and expand on theory about organizational determinants of implementation effectiveness.

## Competing interests

The authors declare that they have no competing interests.

## Author contributions

BJW conceived the idea for the manuscript. RT took the lead in drafting it. BJW, RT, DB, and MJ conducted the research that informed the manuscript, participated in data analysis and interpretation, made editorial and substantive changes to manuscript drafts, and approved the final manuscript. All authors contributed equally to this work. All authors read and approved the final manuscript.
